# Childhood adversity and suicidal ideation in older Korean adults: unraveling the mediating mechanisms of mental health, physical health, and social relationships

**DOI:** 10.1186/s12888-024-05919-5

**Published:** 2024-07-02

**Authors:** Jin-kyung Lee, Jinhee Lee, Moo-Kwon Chung, Taeksoo Shin, Ji Young Park, Kyoung-Joung Lee, Hyo-Sang Lim, Sangwon Hwang, Erdenebayar Urtnasan, Yongmie Jo, Min-Hyuk Kim

**Affiliations:** 1 Institute for Poverty Alleviation and International Development, Yonsei University, Mirae Campus, Wonju, Republic of Korea; 2 Department of Psychiatry, Wonju College of Medicine, Yonsei University, Wonju, Republic of Korea; 3 Department of Global Public Administration, Yonsei University, Mirae Campus, Wonju, Republic of Korea; 4 Department of Business Administration, Yonsei University, Mirae Campus, Wonju, Republic of Korea; 5 Department of Social Welfare, Sangji University, Wonju, Republic of Korea; 6 Department of Biomedical Engineering, Yonsei University, Mirae Campus, Wonju, Republic of Korea; 7 Department of Computer & Telecommunications Engineering, Yonsei University, Mirae Campus, Wonju, Republic of Korea; 8 Department of Precision Medicine, Wonju College of Medicine, Yonsei University, Wonju, Republic of Korea; 9 Artificial Intelligence Bigdata Medical Center, Wonju College of Medicine, Yonsei University, Wonju, Republic of Korea

**Keywords:** Older adults, Suicidal ideation, Adverse childhood experiences, Mediation, Mental health, Physical health, Social relationships

## Abstract

**Background:**

Suicide rates in older adults are much higher than those in younger age groups. Given the rapid increase in the proportion of older adults in Korea and the high suicide rate of this age group, it is worth investigating the mechanism of suicidal ideation for older adults. Generally, adverse childhood experiences are positively associated with suicidal ideation; however, it is not fully understood what mediating relationships are linked to the association between these experiences and current suicidal ideation.

**Methods:**

The data from 685 older Korean adults were analyzed utilizing logistic regression, path analyses, and structural equation modeling. Based on our theoretical background and the empirical findings of previous research, we examined three separate models with mental health, physical health, and social relationship mediators. After that, we tested a combined model including all mediators. We also tested another combined model with mediation via mental health moderated by physical health and social relationships.

**Results:**

The univariate logistic regression results indicated that childhood adversity was positively associated with suicidal ideation in older adults. However, multivariate logistic regression results demonstrated that the direct effect of childhood adversity became nonsignificant after accounting all variables. Three path models presented significant mediation by depression and social support in the association between childhood adversity and suicidal ideation. However, combined structural equation models demonstrated that only mediation by a latent variable of mental health problems was statistically significant. Social relationships moderated the path from mental health problems to suicidal ideation.

**Conclusions:**

Despite several limitations, this study has clinical implications for the development of effective strategies to mitigate suicidal ideation. In particular, effectively screening the exposure to adverse childhood experiences, early identification and treatment of depressive symptoms can play a crucial role in weakening the association between childhood adversity and suicidal ideation in older adults.

**Supplementary Information:**

The online version contains supplementary material available at 10.1186/s12888-024-05919-5.

## Introduction

Globally, 703,000 people die by suicide every year [1] and suicidal behaviors are higher in older adults than in other age groups [2, 3]. In particular, South Korea showed the highest elderly suicide rate (54.8 per 100 000) among the members of the Organization for Economic Cooperation and Development (OECD), and the rate was estimated to be approximately triple the OECD’s average (18.4 per 100 000). As the proportion of elderly people has risen sharply, suicide in the elderly, alongside its enormous economic burden, is expected to become a serious social issue [4]. Previous research has shown that suicidal desire in older adults is more dangerous than in other populations. This population, who are more likely to experience isolation, generally tends to retain positive attitudes toward life but once they are gripped by suicidal ideation, even the first suicidal behavior is likely to involve lethal methods, making them more likely to be found later and vulnerable to death [5]. Given the higher likelihood of developing suicidal ideation and behaviors among older adults, identifying risk factors and underlying mechanisms is crucial to developing strategies to prevent suicidal thoughts in older people.

It is well-known that adverse childhood experiences significantly increase suicidality. Recent meta-analyses reviewing international studies demonstrated that adverse childhood experiences have been associated with suicidality including suicidal ideation, attempts, and events [6–11]. Academically, suicidal ideation is defined as passive or active thoughts about death of oneself, and a suicidal attempt indicates a self-injurious behavior based on suicidal ideation [12]. A suicidal event occurs when a self-injurious attempt with suicidal ideation worsens and causes an emergent situation [12]. As Van Orden et al. [13] summarized, it is difficult to investigate suicidal attempts in a community sample due to the low rate of suicidal attempts among the whole population, the high possibility of exclusion from clinical trials due to safety concerns, and the unavailability of psychological assessments for those who died by suicide. However, although suicidal ideation is not always linked to lethal suicidal attempts, experiencing suicidal ideation significantly increases the risk of suicidal attempts and, in fact, all suicidal attempts are predicated on the existence of prior suicidal ideation. Among clinical cases, it is not difficult to see that persistent suicidal ideation increases the likelihood of recurrent suicidal attempts even when a prior attempt has been successfully halted. Thus, to effectively prevent a completed suicide, an investigation of one’s manner of suicidal ideation would provide useful information in creating effective and meaningful policies.

Thus far, whereas much of the research on adverse childhood experiences and suicidality has focused on adolescents and young adults, there is a growing body of research showing the association between adverse childhood experiences and suicidal ideation throughout an individual’s life [14–17]. Theoretically, the interpersonal theory of suicide [18] explains that individuals with adverse childhood experiences feel burdensome to their family from an early developmental stage and struggle to develop a strong sense of belonging with their parents due to child maltreatment. Sachs-Ericsson et al. [14] proposed that adverse childhood experiences may increase the risk for suicide in late adulthood via several mechanisms, such as biological processes, psychiatric and health comorbidities, and psychosocial development.

According to a recent white paper on suicide prevention in South Korea [19], suicide rates tend to increase with age. Overall, the suicide rate in those 80 and older was the highest at 61.3 per 100 000, 3 times the OECD average in the corresponding age group. The suicide rate for ages 70–79 was the second highest at 41.8 per 100 000, 2.5 times higher than the corresponding OECD average statistics. The suicide rate for ages 60–69 was relatively lower at 28.4 per 100 000, 2.1 times higher than the corresponding statistics for the OECD [19]. Given that there is a significant time between exposure to adverse childhood experiences and suicide in late adulthood, and that adverse childhood experiences influence the overall developmental processes across an individual’s life, it would be worthwhile to explore which mediators play a significant role in the relationship between adverse childhood experiences and suicidal ideation. Given that adverse childhood experiences influence suicidal ideation, the ideal solution would be the prevention of such experiences in the first place. However, the occurrences of adverse childhood experiences are often uncontrollable, which signifies the existence of individuals at high risk for suicidality. Then, the primary concern in an intervention policy should be on figuring out significant mediators and breaking the chain. To date, several previous studies have proposed that various factors could be mediators in the association between adverse childhood experiences and suicidality, ranging from mental disorders (e.g. depression, anxiety) and physical health problems (e.g. cancer, cerebrovascular disease) to social relationships (e.g. social support) [15, 16, 20–33]. However, these studies tend to focus on only a few mediating variables fallen in one specific area rather than examining them across mental, physical, social areas and comparing their effects to find the most significant mediators. To design efficient and effective policies to reduce suicide, we need further research to explore which mediators are more relevant in the mechanism of suicidal ideation amongst a high-risk population. Furthermore, while the suicide rate is highest among older adults and tends to rise with age, previous studies have predominantly concentrated on young or middle adulthood [15, 16, 20–33]. Thus, the specific factors that mediate the pathway from childhood adversity to suicidality in older adults remain incompletely understood. Given that risk factors of suicidal ideation can be different by age, an integrative investigation to figure out significant mediators in older adults would fill the knowledge gap and help to draw policy implications for effective interventions to reduce the rate of suicide amongst older adults.

Regarding the development of suicidal thoughts and behaviors in older adults, the interpersonal theory of suicide [13, 18] provides a persuasive perspective with numerous clinical examples. According to the theory [13, 18], “thwarted belongingness” and “perceived burdensomeness” are two critical factors that significantly trigger suicidal ideation. This theory especially focuses on explaining the mechanism of suicidal behaviors, but it is invaluable for understanding the development of suicidal ideation. Although this theory highlights the merits of an integrated view to enhance our understanding of suicidality, unfortunately, there has not been enough empirical research conducted thus far considering all these critical factors together. In explaining further mechanisms between childhood adversity and suicidal ideation in older adults, we will investigate three mediation models covering mental health, physical health, and social relationship areas based on previous research [14, 34–39]. Our attention will be given to variables connected to thwarted belongingness and perceived burdensomeness among older adults. From our perspective, mental health problems contribute to a distorted perception which leads them to view their circumstances more negatively and increases thoughts such as believing one has no one to rely on (thwarted belongingness) and everyone would be happier if one was gone (perceived burdensomeness). Experiencing physical health problems requires expensive costs and presents many restrictions on their independence, which in turn can increase their perceived burdensomeness. Also, restricted physical and financial independence as consequences of poor health conditions presents a barrier to their active participation in social activities as much as other healthy individuals. Even when making social plans with friends, older adults with poor health conditions often experience situations in which they have to cancel due to their deteriorated health conditions. This can increase the sense of thwarted belongingness. Poor social relationships intensify the effects of social isolation, which increases thwarted belongingness as well. Poor social relationships increase the likelihood of a history of serious relational conflicts or experiences of neglect with others. Therefore, others may perceive the task of meeting the aged population’s basic needs as a serious burden. This increases older adults’ perceived burdensomeness.

Taken together, this research aims to examine mental health, physical health, and social relationships as mediators of the positive association between adverse childhood experiences and suicidal ideation among older adults. The four major hypotheses investigated in this research are shown below:

### Hypothesis 1.1

Older adults with adverse childhood experiences are likely to have suicidal ideation than those without adverse childhood experiences.

### Hypothesis 1.2

Several characteristics such as depressive symptoms, anxiety, and binge drinking that affect mental health mediate the association between adverse childhood experiences and suicidal ideation in older adults.

### Hypothesis 1.3

Physical health problems such as cardiovascular disease or cancer mediate the association between adverse childhood experiences and suicidal ideation in older adults.

### Hypothesis 1.4

Social relationships, including the range of social networks and the degree of perceived social support, mediate the association between adverse childhood experiences and suicidal ideation in older adults.

## Methods

### Data

Data collected from 685 older Korean adults were analyzed. Participants were required to be at least 55 years old and not suffer from substance use disorder, dementia, or serious illness in order to complete a face-to-face interview for 90 min. The participants were recruited from the large national cohort study, the Korean Genome and Epidemiology Study-Cardiovascular Disease Association Study (KoGES_CAVAS), via phone. The KoGES_CAVAS was a multi-sited study with approximately 28,500 adults in South Korea. Among the KoGES_CAVAS, 3,620 adults lived in Wonju, a large local community mixed with urban and rural areas. Out of 3,620 adults, 1,896 people changed or did not report available cell phone numbers. Out of 1,724 adults who reported available cell phone numbers, we recruited 685 participants after excluding 1,039 people who did not meet the inclusion criteria. Different from KoGES_CAVAS, we designed new semi-structured interviews to collect data about older adults’ mental health from the participants in order to understand the mechanisms of late-life depression [40]. After agreeing to participate in this semi-structured interview, the participants visited a campus once from December 2020 to April 2021. After being provided an explanation of this study, they voluntarily signed the written consent form. Subsequently, they had a 1:1 session with a trained researcher to complete the interview. Data about participants’ demographic characteristics, close relationships, and physical and mental health were collected during this interview.

### Measures

#### Childhood adversity

The severity of adverse childhood experiences was measured by the short form of the Early Trauma Inventory [41]. This scale asked about each participant’s general traumas, physical punishment, emotional abuse, and sexual events before the age of 18. In this study, a total score was constructed from 27 binary items such as “Did you ever witness violence towards others, including family members?”, “Were you ever slapped in the face with an open hand?”, and “Were you often put down or ridiculed?” (Cronbach’s alpha = 0.75).

#### Suicidal ideation

Suicidal ideation in older adults was measured by the last item of the Patient Health Questionnaire-9 (PHQ-9) [42]. This item asked how often a participant had been bothered over the past two weeks by thoughts that they would be better off dead or hurting themselves in some way. Original responses ranged from “0 Not at all” to “3 Nearly every day”. When delineating suicidal ideation, the contrast between never experiencing a suicidal thought and experiencing one is qualitatively distinct, unlike variations in the frequency of suicidal thoughts. Original responses indicating “Not at all” were recoded as 0 while responses of “Several days”, “More than half the days”, and “Nearly every day” were all recoded as 1.

#### Mental health problem mediators

Depression, anxiety, and binge drinking were tested as mediators related to mental health functioning. The degree of depressive symptoms (PHQ-8) was constructed by aggregating eight items (Cronbach’s alpha = 0.76) from the PHQ-9 [42] with the exclusion of the last item regarding suicidal ideation. Under the prompt “Over the last two weeks, how often have you been bothered by any of the following problems?”, eight items including “Little interest or pleasure in doing things” and “Feeling down, depressed, or hopeless” were asked to each participant. Each item ranged from 0 for “Not at all” to 3 for “Nearly every day”.

Next, anxiety was measured by the General Anxiety Disorder-7 screening tool (GAD-7) [43]. Under the prompt “Over the last two weeks, how often have you been bothered by the following problems?”, seven items including “Trouble relaxing” and “Worrying too much about different things” were asked to each participant. Each item was scored from 0 for “Not at all” to 3 for “Nearly every day”. A sum score was created using seven items (Cronbach’s alpha = 0.87).

Last, binge drinking was defined as consuming 5 or more drinks on an occasion based on the definition by the Centers for Disease Control and Prevention [44]. It was coded as a binary variable (1 = binge drinking, 0 = no).

#### Physical health problem mediators

The presence of six common physical health problems was determined based on the report of ‘having been diagnosed by the doctor’ from participants. High blood pressure, hyperlipidemia, diabetes mellitus, cardiovascular disease, cerebrovascular accident, and cancer were coded as a binary variable to indicate if a participant had ever been diagnosed with the disease (1 = yes, 0 = no).

#### Social relationship mediators

Social networks and perceived social support were tested as social relationship mediators. Social support was constructed by averaging 12 items from the Multidimensional Scale of Perceived Social Support [45](Cronbach’s alpha = 0.92). The items of social support included “There is a special person who is around when I am in need”, “I get the emotional help and support I need from my family”, and “I can count on my friends when things go wrong”. Each item was scored from “1 Strongly disagree” to “5 Strongly agree”.

The variable of social networks was constructed by aggregating 18 items from the Lubben Social Network Scale [46](Cronbach’s alpha = 0.85). The items of social networks included “How many relatives do you see or hear from at least once a month?”, “How many friends do you feel at ease with that you can talk about private matters?”, and “How many neighbors do you feel close to such that you could call on them for help?”. Each item was scored from 0 to 5 in which a higher score indicated a wider social network and more frequent contacts with others.

#### Covariates

Sex was coded as 1 for males and 0 for females. Age was coded as a continuous variable with an ordinal scale. Participants in their 50s were coded as 1, those in 60s were coded as 2, those in 70s were coded as 3, and those in 80s were coded as 4. Education was also coded with an ordinal scale. Participants who completed elementary school or had a lower education level were coded as 1, those who completed middle school or high school were coded as 2, and those with a community college or higher education level were coded as 3. In terms of marital status, married participants were coded as 1 and unmarried participants were coded as 0. Household monthly income was reported in the official currency of South Korea (Won). Log transformation was applied for household income to adjust for its high skewness.

### Statistical analysis

As a preliminary analysis, we first checked descriptive statistics including means, standard deviations, frequencies, and proportions for all variables. For group comparison by suicidal ideation, independent t-tests and chi-square tests were performed. We also ran multicollinearity test, but we did not find any problematic issues (mean VIF = 1.31, minimum VIF = 1.04, maximum VIF = 1.93).

As main analyses, univariate and multivariate logistic regression models were tested to estimate crude effects and adjusted effects of the independent variables on suicidal ideation. Given that the number of individuals with suicidal ideation is small in our data, we conducted sensitivity analyses with the same set of all the variables in the multivariate logistic regression, and the results are presented in the Appendix.

According to the literature [14, 34–39], three path models regarding mental health, physical health, and social relationships were tested with different sets of mediators. Based on the empirical evidence that depression, anxiety, and alcohol use disorders significantly mediated the relationship between childhood adversity and suicidal ideation [34], the first path model tested three mediators (depression, anxiety, binge drinking) that were related to mental health dysfunctions. Given that childhood adversity is associated with the occurrence of physical health problems [35, 47] and physical health problems increase suicidal ideation in adulthood [36], the second path model consisted of six mediators (high blood pressure, hyperlipidemia, diabetes mellitus, cardiovascular disease, cerebrovascular accident, and cancer) that were common physical health problems among older Korean adults. Based on the previous research demonstrating that social relationships significantly mediated the association between childhood adversity and suicidal ideation [20, 38, 39], the third path model investigated the role of two mediators (social support, social network) that represented the quality and quantity of social relationships.

All three mediation models had childhood adversity as an exogenous variable and suicidal ideation as the last endogenous variable. To make it easy to compare this research with other research investigating mediators, we tested mediation paths through each mediator variable within three separate models in adherence with previous research. Covariates such as sex, age, education, household income, and marital status were controlled for all endogenous variables. For mediation models, bootstrapping with 5,000 iterations [48–50] was applied.

Since we are interested in comprehensively investigating the mediation mechanisms of suicidal ideation for older adults, we also tested a combined model by integrating all mediators across three models. For better visibility, we present the combined model in which three latent mediator variables (mental health problems, physical health problems, and social relationships) were constructed. Considering the insufficient factor loadings of the six binary physical health problems, only the latent variable of physical health problems was replaced with the observed continuous variable, indicating the number of physical health problems of a participant. Contrary to our expectation, binge drinking showed a non-significant path to explain suicidal ideation and a poor factor loading of a latent variable of mental health problems. We excluded binge drinking as an indicator of the latent mediator in this combined model.

After testing simple mediation paths in the combined model, we also explored the joint effects to see if the mediation by mental health problems differed by physical health problems or social relationships. Thus, we changed two nonsignificant mediators of physical health problems and social relationships into moderators. We added their interactions with mental health problems on the path explaining suicidal ideation in this combined model.

For model fit indices in three path models and two structural equation models, we reported frequently used model fit indices [51–53]: Chi-Squares (χ^2^), root mean square errors of approximation (RMSEA), comparative fit indexes (CFI), and standardized root mean square residuals (SRMR). To indicate a good model fit, it is recommended to have nonsignificant χ^2^ [51], RMSEA ≤ 0.07 [52], CFI ≥ 0.95 [53], and SRMR ≤ 0.08 [53]. STATA 17.0 for descriptive statistics and logistic regressions, Python for sensitivity analyses, and Mplus 8.9 for path and structural equation models were used.

## Results

### Descriptive statistics

Table 1 indicates the descriptive statistics of the sample. Out of 685 older adults, 31 people (4.53%) reported that they had experienced suicidal ideation during the worst two-week period in the past month. On average, people with suicidal ideation tended to report higher scores in childhood adversity, depressive symptoms, and anxiety than others. There was little difference in binge drinking between people with vs. without suicidal ideation. Among the whole sample, approximately 17% reported consuming 5 or more cups of alcohol in one setting. 301 participants (43.94%) reported they had been diagnosed with high blood pressure, and 267 participants (38.98%) reported they had ever had hyperlipidemia. 140 participants (20.44%) reported they had experienced diabetes mellitus, and 89 participants (12.99%) reported they had suffered from cardiovascular disease. 72 participants (10.51%) reported they had been diagnosed with cancer, and 37 participants (5.40%) reported they had experienced cerebrovascular accidents. Older adults with suicidal ideation showed higher incidence rates in all six of these health issues compared to those without suicidal ideation. However, older adults with suicidal ideation reported lower average scores in social networks and perceived social support than those without suicidal ideation. In the group with suicidal ideation, the proportions of female, elderly, and lower education level individuals were higher than those in the group without suicidal ideation. Additionally, the group with suicidal ideation reported lower household income than the group without suicidal ideation.

**Table 1:** Descriptive statistics

	Total (*N* = 685)	With suicidal ideation (*n* = 31)	Without suicidal ideation (*n* = 654)	Independent t-test (t) or chi-square test (χ^2^)
Suicidal ideation	N (%)	N (%)	N (%)	
Yes	31 (04.53%)	31 (100.0%)	-	
No	654 (95.47%)	-	654 (100.0%)	
	M (SD)	M (SD)	M (SD)	
Adverse childhood experiences	2.85 (2.95)	4.32 (3.49)	2.78 (2.90)	t = -2.87^**^
Depressive symptoms	0.33 (0.42)	1.07 (0.68)	0.29 (0.37)	t = -10.81^***^
Anxiety	1.38 (2.76)	6.03 (5.19)	1.16 (2.38)	t = -10.33^***^
Binge drinking	N (%)	N (%)	N (%)	t = 0.14
Yes	117 (17.08%)	5 (16.13%)	112 (17.13%)	
No	568 (82.92%)	26 (83.87%)	542 (82.87%)	
High blood pressure	N (%)	N (%)	N (%)	t = -1.25
Yes	301 (43.94%)	17 (54.84%)	284 (43.43%)	
No	384 (56.06%)	14 (45.16%)	370 (56.57%)	
Hyperlipidemia	N (%)	N (%)	N (%)	t = -3.77***
Yes	267 (38.98%)	22 (70.97%)	245 (37.46%)	
No	418 (61.02%)	9 (29.03%)	409 (62.54%)	
Diabetes mellitus	N (%)	N (%)	N (%)	t = -3.05**
Yes	140 (20.44%)	13 (41.94%)	127 (19.42%)	
No	545 (79.56%)	18 (58.06%)	527 (80.58%)	
Cardiovascular disease	N (%)	N (%)	N (%)	t = -2.73**
Yes	89 (12.99%)	9 (29.03%)	80 (12.23%)	
No	596 (87.01%)	22 (70.97%)	574 (87.77%)	
Cerebrovascular accident	N (%)	N (%)	N (%)	t = -2.71**
Yes	37 (05.40%)	5 (16.13%)	32 (04.89%)	
No	648 (94.60%)	26 (83.87%)	622 (95.11%)	
Cancer	N (%)	N (%)	N (%)	t = -0.44
Yes	72 (10.51%)	4 (12.90%)	68 (10.40%)	
No	613 (89.49%)	27 (87.10%)	586 (89.60%)	
	M (SD)	M (SD)	M (SD)	
Social support	3.92 (0.70)	3.31 (0.93)	3.95 (0.68)	t = 5.02^***^
Social network	42.76 (13.18)	35.97 (14.61)	43.09 (13.04)	t = 2.95^**^
Sex	N (%)	N (%)	N (%)	t = 1.16
Male	290 (42.34%)	10 (32.26%)	280 (42.81%)	
Female	395 (57.66%)	21 (67.74%)	374 (57.19%)	
Age	N (%)	N (%)	N (%)	χ^2^ = 4.96
50s	68 (09.93%)	2 (06.45%)	66 (10.09%)	
60s	317 (46.28%)	12 (38.71%)	305 (46.64%)	
70s	240 (35.04%)	11 (35.48%)	229 (35.02%)	
80s	60 (08.76%)	6 (19.35%)	54 (08.26%)	
Education	N (%)	N (%)	N (%)	χ^2^ = 10.64**
≤ Elementary	200 (29.20%)	17 (54.84%)	183 (27.98%)	
Middle or high	319 (46.57%)	8 (25.81%)	311 (47.55%)	
≥ College (2-3yrs or 4yrs)	166 (24.23%)	6 (19.35%)	160 (24.46%)	
Marital status	N (%)	N (%)	N (%)	t = 2.81**
Married	612 (89.34%)	23 (74.19%)	589 (90.06%)	
Unmarried	73 (10.66%)	8 (25.81%)	65 (09.94%)	
	M (SD)	M (SD)	M (SD)	
Household income (Won; monthly)	2,912,381 (2,288,407)	2,141,290 (1,645,286)	2,948,931 (2,309,001)	t = 1.92

^*^p < .05, ^**^p < .01, ^***^p < .001

### Logistic regression predicting suicidal ideation

Table 2 indicates the crude and adjusted effects of all independent variables on suicidal ideation. Examination of the crude effects revealed childhood adversity (Crude OR = 1.15, *p* < .01), depressive symptoms (Crude OR = 9.71, *p* < .001), anxiety (Crude OR = 1.32, *p* < .001), hyperlipidemia (Crude OR = 4.08, *p* < .001), diabetes mellitus (Crude OR = 3.00, *p* < .01), cardiovascular disease (Crude OR = 2.94, *p* < .01), and cerebrovascular accident (Crude OR = 3.74, *p* < .05) to be positively associated with suicidal ideation. As expected, social support (Crude OR = 0.36, *p* < .001) and social network (Crude OR = 0.96, *p* < .01) were negatively associated with suicidal ideation. However, in the adjusted model after accounting for all independent variables, only the direct effects of depressive symptoms (adjusted OR = 4.91, *p* < .01) and anxiety (adjusted OR = 1.14, *p* < .05) among key variables remained statistically significant in explaining suicidal ideation.

**Table 2:** Crude and adjusted effects on suicidal ideation

Y = Suicidal ideation		
	Crude OR (95% CI)	Adj. OR (95% CI)
Adverse childhood experiences	1.15^**^ (1.04, 1.26)	1.03 (0.90, 1.18)
Depressive symptoms	9.71^***^ (5.22, 18.06)	4.91^**^ (1.83, 13.13)
Anxiety	1.32^***^ (1.22, 1.43)	1.14^*^ (1.00, 1.30)
Binge drinking	0.93 (0.35, 2.48)	1.07 (0.28, 4.07)
High blood pressure	1.58 (0.77, 3.26)	0.93 (0.35, 2.43)
Hyperlipidemia	4.08^***^ (1.85, 9.01)	2.38 (0.89, 6.33)
Diabetes mellitus	3.00^**^ (1.43, 6.28)	1.68 (0.66, 4.29)
Cardiovascular disease	2.94^**^ (1.31, 6.60)	1.21 (0.41, 3.53)
Cerebrovascular accident	3.74^*^ (1.35, 10.38)	2.77 (0.73, 10.48)
Cancer	1.28 (0.43, 3.76)	1.61 (0.47, 5.57)
Social support	0.36^***^ (0.24, 0.56)	1.03 (0.53, 2.02)
Social network	0.96^**^ (0.93, 0.99)	0.97 (0.94, 1.02)
Age	1.52 (0.97, 2.39)	1.65 (0.88, 3.12)
Male	0.64 (0.29, 1.37)	1.02 (0.33, 3.12)
Education	0.53^*^ (0.31, 0.90)	0.50^*^ (0.26, 0.96)
Married	0.32^**^ (0.14, 0.74)	0.58 (0.18, 1.89)
Household income	0.97 (0.83, 1.15)	1.24 (0.88, 1.74)
Constant	-	0.00^*^ (0.00, 0.25)
R^2^	-	0.341

^*^p < .05, ^**^p < .01, ^***^p < .001

### Path model with mental health problem mediators

**Fig. 1:**
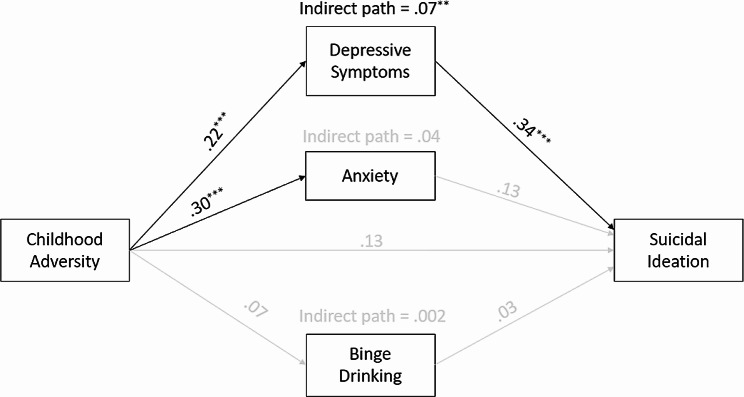
Path results with mental health problem mediators ^*^p < .05, ^**^p < .01, ^***^p < .001 Note: A black line indicates a statistically significant path (*p* < .05) while a gray line indicates a nonsignificant path. Covariates such as age, sex, education, marital status, and household income were controlled for all endogenous variables

The results from the first path model with mental health mediators are shown in Fig. 1. This was a saturated model, so the model fit indices were *χ*^*2*^(*df*) = 718.04(30), *p* = .000, RMSEA = 0.000, CFI = 1.000, and SRMR = 0.002. When mediation paths by depression, anxiety, and binge drinking were tested, the results demonstrated that childhood adversity was positively associated with depression (*β* = 0.22, *p* < .001), and depression was positively associated with suicidal ideation (*β* = 0.34, *p* < .001). The indirect effect of childhood adversity via depression was statistically significant in explaining suicidal ideation (*β* = 0.07, *p* < .01). In terms of anxiety, childhood adversity was significantly associated with anxiety in late adulthood (*β* = 0.30, *p* < .001) but anxiety was not significantly associated with suicidal ideation. Different from our hypothesis, childhood adversity was not statistically significant in predicting binge drinking, and binge drinking was not significantly associated with suicidal ideation. When including all these mediation paths, the direct effect of childhood adversity became statistically nonsignificant in explaining suicidal ideation.

### Path model with physical health problem mediators

**Fig. 2:**
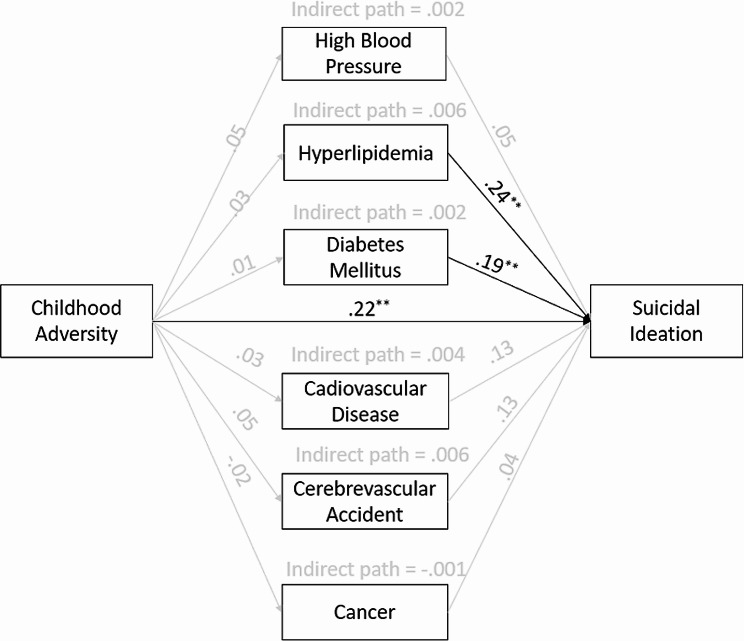
Path results with physical health problem mediators ^*^p < .05, ^**^p < .01, ^***^p < .001 Note: A black line indicates a statistically significant path (*p* < .05) while a gray line indicates a nonsignificant path. Covariates such as age, sex, education, marital status, and household income were controlled for all endogenous variables

The results from the second path model with physical health mediators are given in Fig. 2. As expected, childhood adversity was positively associated with suicidal ideation (*β* = 0.22, *p* < .001). Furthermore, older adults who had hyperlipidemia (*β* = 0.24, *p* < .01) and those with diabetes mellitus (*β* = 0.19, *p* < .01) were more likely to experience suicidal ideation. However, childhood adversity was not significantly associated with any of the six common physical health problems (high blood pressure, hyperlipidemia, diabetes mellitus, cardiovascular disease, cerebrovascular accident, and cancer). Therefore, none of the physical health problems played an important mediating role in explaining the relationship between childhood adversity and suicidal ideation. In this model, CFI had a poor fit but other model fit indices were acceptable (*χ*^*2*^(*df*) = 94.39(63), *p* = .006, RMSEA = 0.027, CFI = 0.762, and SRMR = 0.047).

### Path model with social relationship mediators

**Fig. 3:**
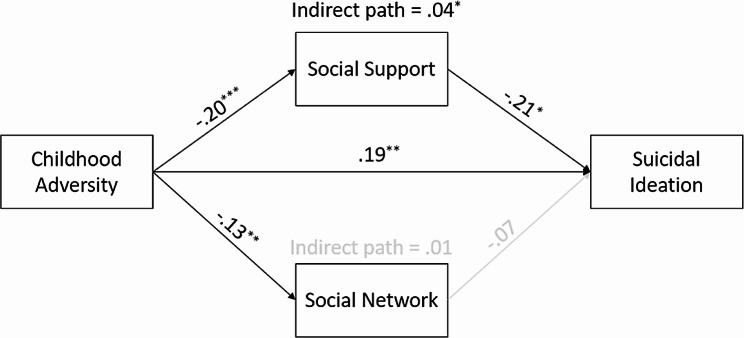
Path results with social relationship mediators ^*^p < .05, ^**^p < .01, ^***^p < .001 Note: A black line indicates a statistically significant path (*p* < .05) while a gray line indicates a nonsignificant path. Covariates such as age, sex, education, marital status, and household income were controlled for all endogenous variables

The results from the third path model with social relationship mediators are presented in Fig. 3. Model fit indices indicated that this was a saturated model (*χ*^*2*^(*df*) = 280.97(21), *p* = .000, RMSEA = 0.000, CFI = 1.000, and SRMR = 0.000). As anticipated, childhood adversity was negatively associated with social support (*β* = − 0.20, *p* < .001) and social support was negatively associated with suicidal ideation (*β* = − 0.21, *p* < .05). The indirect effect of childhood adversity on suicidal ideation via social support was statistically significant (*β* = 0.04, *p* < .05). However, although childhood adversity was significantly associated with social networks (*β* = − 0.13, *p* < .01), social network was not significantly associated with suicidal ideation. This demonstrated that the quality of perceived social support was more important than the quantity of social networks in explaining suicidal ideation.

### Combined structural equation models

In addition, a structural equation model was tested after constructing a latent mediator variable with corresponding observed variables for each model (i.e. mental health model, physical health model, and social relationship model) and including all these three latent variables within the model. As mentioned in the Methods section, due to poor factor loadings, binge drinking was excluded from the latent variable of mental health characteristics. Also, the latent variable of physical health mediators was replaced with the continuous variable of the number of physical illnesses. All covariates such as age, gender, education, marital status, and household income were controlled for physical health, mental health, and social relationship mediators and the outcome variable (suicidal ideation) in this combined model. Model fit indices for this combined model with three mediators indicated a good model fit (RMSEA = 0.064, CFI = 0.948, SRMR = 0.045).

In the combined model, only the latent mediator variable of mental health problems was significantly explained by childhood adversity (ACE) and did significantly explain suicidal ideation (SI) in late adulthood (ACE → mental health problems: *β* = 0.33, *p* < .001; mental health problems → SI: *β* = 0.44, *p* < .001). The observed continuous mediator of the number of physical health problems was significantly associated with suicidal ideation, but it was not significantly explained by childhood adversity (ACE → physical health problems: *β* = 0.05, *p* = .238; physical health problems → SI: *β* = 0.26, *p* < .001). On the contrary, childhood adversity was significantly associated with the latent mediator variable of social relationships, but this latent social relationship variable was statistically nonsignificant in explaining suicidal ideation in late adulthood (ACE → social relationships: *β* = − 0.21, *p* < .001; social relationships → SI: *β* = − 0.12, *p* = .129).

**Fig. 4:**
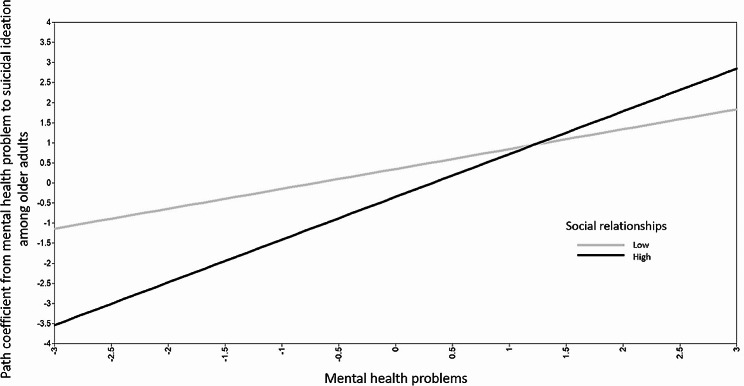
Moderation by social relationships on the path from mental health problems to suicidal ideation

Next, we also tested moderated mediation paths in a structural equation model by changing two nonsignificant mediator variables representing physical health problems and social relationships into moderator variables. We added their interactions with mental health (i.e., mental health problems x physical health problems, mental health problems x social relationships) on the path predicting suicidal ideation in the combined model. By doing this, we were interested to see if the mediation via mental health differed by physical health problems or social relationships. In this latent interaction model, social relationships significantly moderated the effects of mental health problems on suicidal ideation (mental health problems x social relationships → SI: *β* = 0.12, *p* < .01) while the interaction between mental health problems and physical health problems was not statistically significant. The significant interaction plot between two latent variables (mental health problems x social relationships) was presented in Fig. 4.

## Discussion

This study investigates the direct and indirect associations between childhood adversity and suicidal ideation in community-dwelling older adults in South Korea. The results of the present study demonstrate that older adults with adverse childhood experiences were more likely to experience suicidal ideation. However, mental health problems, especially depression explained the path from childhood adversity and suicidal ideation in late adulthood. Social support seemed to mediate the association between childhood adversity and suicidal ideation, but in fact, social relationships did not mediate this path but rather moderated the effect of mental health problems on suicidal ideation, intensifying the effect of mental health problems.

### Direct association between childhood adversity and suicidal ideation

Our findings support the research hypothesis that childhood adversity increases the risk of suicidal ideation in late adulthood. This is in line with the interpersonal theory of suicide [13]. Exposure to adverse childhood experiences is a significant risk factor of suicidal ideation because people who were previously exposed to more adverse childhood experiences are likely to be more sensitive to developing feelings of burdensomeness and thwarted belongingness in a given situation [13, 18]. Our findings demonstrate that childhood adversity not only triggers the development of suicidal ideation at an earlier developmental period in one’s life, but also increases the likelihood of suicidal ideation later in life. One clinical implication of these results is that screening the exposure of adverse childhood experiences should be a crucial component of effective strategies for suicide prevention. As shown in the recent scoping review [54], more discussion is needed about which measures or what aspects of adverse childhood experiences are more relevant to preventing suicidality. Previous research has discussed that it is difficult to separate and estimate the unique effect of each specific type of adverse childhood experiences since child sexual, physical, emotional, and general abuse often co-occur within a household over a long period [14]. In this study, the types of adverse childhood experiences were quantified for each participant. This is one viable approach.

On the other hand, the findings from our combined model that the direct effect of childhood adversity became nonsignificant after including mediation paths instill hope. In other terms, if we successfully intervene to break the mediation chain, the risk of suicidal ideation caused by childhood adversity would be significantly reduced. Further research is needed to fully reveal the mechanism of how suicidal ideation is influenced by childhood adversity, particularly among those who were exposed to greater adverse childhood experiences. To comprehensively investigate the mechanism from childhood adversity to suicidal ideation in late adulthood, future research requires longitudinal data collection from a larger representative sample.

### Indirect association between childhood adversity and suicidal ideation through mental health mediators

This study supports the positive association between adverse childhood experiences and suicidal ideation through mental health mediators. Previous studies using a variety of methods (including longitudinal and cross-sectional studies) have demonstrated that depression and anxiety have a mediating role in the relationship between adverse childhood experiences and suicidality [25–27, 29]. Our results partially supported it by demonstrating that depression is a significant mediator in this relationship. Our findings suggest that early identification and treatment of depression is very important for the prevention of suicidal ideation in older Korean adults.

In terms of anxiety, the results of the present study demonstrate that the positive association between childhood adversity and anxiety was statistically significant, but the association between anxiety and suicidal ideation was weaker than we anticipated. Given the high factor loading of anxiety to construct the latent mental health variable, and the latent variable’s significant mediation in the combined model, we interpret that heightened anxiety influenced by childhood adversity would be a contextual factor (i.e. worsening the risk of suicidal ideation with depressive symptoms) rather than directly increasing suicidal ideation. Considering the high association between childhood adversity and anxiety in late adulthood, early diagnosis and clinical treatments for anxiety disorder would be effective for those who had adverse childhood experiences.

Contrary to popular belief, binge drinking was not significantly associated with adverse childhood experiences or suicidal ideation. Most previous research has reported that adverse childhood experiences are associated with alcohol abuse later in life, including alcohol dependence or binge drinking, which affects suicidal behaviors [10, 15, 22, 25, 27, 55]. However, in our study, binge drinking was not associated with either adverse childhood experiences nor with suicidal ideation. This is in line with another previous research reporting nonsignificant mediation by alcohol misuse [38]. One thing we were concerned was that our participants, especially men, might have reported less alcohol use than the general Korean population [56]. It is not certain whether our participants consume less alcohol than average or if the participants underreported their consumption, and this could possibly have affected our conclusion. Further study is needed to confirm the role of alcohol use problems in the connections between adverse childhood experiences to suicidal ideation.

### Indirect association between childhood adversity and suicidal ideation through physical health mediators

Our research hypothesis that physical health problems would significantly increase suicidal ideation in older adults is partially supported in this study. When we tested separate mediation paths by six common physical health problems in the path model, only hyperlipidemia and diabetes mellitus were significantly positively associated with suicidal ideation. However, if we tested the mediation by the number of physical health problems in the combined structural equation model, older adults with more health problems were more likely to experience suicidal ideation. This implies that the severity of physical health problems would be more relevant to suicidal ideation in late adulthood than the type of physical health problems. Also, we asked about the history of physical health problems but the current health condition would be more related to suicidal ideation. In our data, the discrepancy between the correlations of the two chronic diseases and other physical health problems concerning suicidal ideation can be explained by the social history of physical health problems, which differs based on the type of disease. For example, the diagnosis of hyperlipidemia and diabetes mellitus was associated with a high risk of feeling like a burden to one’s family. However, in the case of cancer, our participants who have survived cancer were enthusiastic to open their diagnosis and their successful treatment, showing an increase in feelings of gratitude in their lives.

One interesting finding is that childhood adversity was not significantly associated with any history of physical health problems (high blood pressure, hyperlipidemia, diabetes mellitus, cardiovascular disease, cerebrovascular accident, and cancer). Given that adverse childhood experiences influence physical health problems and physical health problems are risk factors for suicide among older adults in previous studies [10, 14, 57], we hypothesized that these chronic diseases would be possible mediators in the path from adverse childhood experiences to suicidal ideation. However, even the number of physical health problems was not predicted by childhood adversity. Our results suggest that the effects of childhood adversity on physical health might involve more complex and individual life factors. Previous studies have suggested various mechanisms: For example, the effects of childhood adversity on physical health can vary depending on the number or types of adverse childhood experiences [10, 11]. It is also likely that the relationships between childhood adversity and the development of chronic diseases differ by sex, disability, severity of pain, or comorbid depression and anxiety [57]. Altogether, these factors would make the relationships between childhood adversity and physical health problems more complex. Future research is recommended to delve into the roles of these various factors and physical health problems between childhood adversity and suicidal ideation.

### Roles of social relationships in the association between childhood adversity and suicidal ideation

The results from the path model with social relationship mediators demonstrate that perceived social support mediated the relationship between childhood adversity and suicidal ideation in late adulthood, but social networks did not. Specifically, more exposure to childhood adversity was associated with poorer social support and weaker social networks. However, weaker social networks were not significantly associated with higher suicidal ideation when poorer social support was. This highlights the importance of social support as a mediator between childhood adversity and suicidal ideation [25, 28, 33]. Future research is recommended to investigate if the underlying mechanism by which social functioning influences suicidal ideation in older adults with adverse childhood experiences is different from the mechanism in those without adverse childhood experiences. If the underlying mechanism is different, more tailored methods of intervention focused on social relationships would need to be developed.

Meanwhile, the new findings from our combined models suggest an interesting possibility that social relationships play a significant role as a moderator in the mediation path from childhood adversity to suicidal ideation through mental health problems. Despite different significance in mediation effects in the basic path model, high factor loadings of perceived social support and social network in the combined structural equation model reflected the fact that the directions of social support and social network are basically similar. The combined structural equation model demonstrated that greater childhood adversity was significantly associated with poorer social relationships, but poorer social functioning was not directly related to heightened suicidal ideation in late adulthood. These are different from the previous research [28, 31] which demonstrated the significant mediation paths by poorer social functioning in the relationship between childhood adversity suicidality among adolescents and young adults. In our combined model, the joint effect between mental health problems and social relationships was noticeable. When a participant had a low risk of mental health problems, a higher level of social functioning was significantly associated with lower suicidal ideation. However, the role of social relationships as a protective factor significantly disappeared when a participant experienced a high level of mental health problems. These results support that the functions of social relationships among older adults would be different from younger populations. Based on the findings, we anticipate the effects of protective factors would be much lower among older adults with a high risk. Further research is required to investigate the role of social relationships, particularly in high-risk older adults experiencing serious mental health problems.

### Limitations

The present study has some limitations. First, this is a cross-sectional study; therefore, all associations reported in this study should be interpreted as tendencies instead of causal relationships. Second, most of the data was measured retrospectively which inherently contains some amount of recall or social desirability bias. Although we interviewed each participant in private to prevent this bias, it still may not be wholly avoidable. Third, we used one item of the PHQ-9 as the outcome variable, suicidal ideation. This question about suicidal ideation captures the short-term suicidal desire of participants at the moment of data collection. Given that suicidal ideation tends to fluctuate over time, this may affect the extent of the association, but we were not able to capture that fully. Fourth, our study had a small sample size, which prevented us from further subgroup analysis stratified by gender, adverse childhood experiences, or subscales of social networks. Further study utilizing longitudinal data with a larger sample size is recommended. Lastly, there might be some other variables that have not been considered in this study but can influence the association between childhood adversity and suicidal ideation.

### Conclusions

Despite several limitations, this study contributes to broader research on the mechanisms of suicidal ideation in older adults. Our data from a community sample indicates that more exposure to childhood adversity is associated with suicidal ideation even in late adulthood. Based on previous research, we tested three different mediation models to explain the association between childhood adversity and suicidal ideation. The major findings of this study support that mental health problems such as depressive symptoms did significantly mediate the effects of childhood adversity on suicidal ideation in late adulthood. Although it is unclear the relationship between childhood adversity and physical health problems, the association between physical health problems and suicidal ideation in late adulthood is supported. The association between social relationships and suicidal ideation was weaker than we anticipated, but childhood adversity was still significant in explaining social relationships in late adulthood. In this study, the role of social relationships among older adults seems to be different from that of younger people, which requires further research. We believe this study makes a meaningful contribution in revealing the mechanisms regarding suicidal ideation in older adults. This study has clinical implications for the development of effective strategies to prevent suicidal ideation among older adults in South Korea. Most notably, screening the degree of adverse childhood experiences, early identification or treatment of mental health problems, and obtaining more information of social relationships in late adulthood can play a critical role in breaking the association between childhood adversity and suicidal ideation in older adults.

### Electronic supplementary material

Below is the link to the electronic supplementary material.

Supplementary Material 1

## Data Availability

The datasets analyzed during the current study are not publicly available due to participants’ confidentiality but are available from the corresponding author on reasonable request.
