# Supplementary material for: Childhood adversity and suicidal ideation in older Korean adults: unraveling the mediating mechanisms of mental health, physical health, and social relationships
Source: BMC Psychiatry. 2024 Jul 2;24:485. doi: 10.1186/s12888-024-05919-5 (PMC11221153; doi:10.1186/s12888-024-05919-5)
Supplement: Supplementary file 1 — Supplementary Material 1 [file 12888_2024_5919_MOESM1_ESM.docx]

Appendix. Sensitivity analysis results.

| Y = Suicidal ideation | Description | Accuracy | F1 score | ROC AUC |
| --- | --- | --- | --- | --- |
| Baseline Model | Logistic regression without adjustments | .97 | .29 | .84 |
| Weighted Model | Logistic regression with class weights | .82 | .16 | .81 |
| Oversampling | SMOTE applied to minority class (Y = 1) | .83 | .17 | .82 |
| Undersampling | Random undersampling of majority class (Y = 0) | .71 | .11 | .77 |
